# Light-dependent magnetoreception in birds: increasing intensity of monochromatic light changes the nature of the response

**DOI:** 10.1186/1742-9994-4-5

**Published:** 2007-02-15

**Authors:** Roswitha Wiltschko, Katrin Stapput, Hans-Joachim Bischof, Wolfgang Wiltschko

**Affiliations:** 1 Fachbereich Biologie der J.W. Goethe-Universität, Siesmayerstraße 70, D-60054 Frankfurt a.M., Germany; 2 Fakultät Biologie, Universität Bielefeld, Lehrstuhl Verhaltensforschung, Postfach 100131, D-35501 Bielefeld, Germany

## Abstract

**Background:**

The Radical Pair model proposes that magnetoreception is a light-dependent process. Under low monochromatic light from the short-wavelength part of the visual spectrum, migratory birds show orientation in their migratory direction. Under monochromatic light of higher intensity, however, they showed unusual preferences for other directions or axial preferences. To determine whether or not these responses are still controlled by the respective light regimes, European robins, *Erithacus rubecula*, were tested under UV, Blue, Turquoise and Green light at increasing intensities, with orientation in migratory direction serving as a criterion whether or not magnetoreception works in the normal way.

**Results:**

The birds were well oriented in their seasonally appropriate migratory direction under 424 nm Blue, 502 nm Turquoise and 565 nm Green light of low intensity with a quantal flux of 8·10^15 ^quanta s^-1 ^m^-2^, indicating unimpaired magnetoreception. Under 373 nm UV of the same quantal flux, they were not oriented in migratory direction, showing a preference for the east-west axis instead, but they were well oriented in migratory direction under UV of lower intensity. Intensities of above 36·10^15 ^quanta s^-1 ^m^-2 ^of Blue, Turquoise and Green light elicited a variety of responses: disorientation, headings along the east-west axis, headings along the north-south axis or 'fixed' direction tendencies. These responses changed as the intensity was increased from 36·10^15 ^quanta s^-1 ^m^-2 ^to 54 and 72·10^15 ^quanta s^-1 ^m^-2^.

**Conclusion:**

The specific manifestation of responses in directions other than the migratory direction clearly depends on the ambient light regime. This implies that even when the mechanisms normally providing magnetic compass information seem disrupted, processes that are activated by light still control the behavior. It suggests complex interactions between different types of receptors, magnetic and visual. The nature of the receptors involved and details of their connections are not yet known; however, a role of the color cones in the processes mediating magnetic input is suggested.

## Background

The Radical Pair model of magnetoreception by Ritz and colleagues [1] proposes that directional information from the geomagnetic field is obtained with the help of radical pair processes taking place in the eye. By photon absorption, molecules are elevated to an excited state, where they form singlet and triplet radical pairs, with the ratio between singlets and triplets depending on the alignment of the respective molecules in the magnetic field. A comparison of, e.g., the triplet yield in the various spatial directions would indicate magnetic North [for details, see [1]]. For birds, this model is now supported by experimental evidence demonstrating the involvement of radical pair mechanisms [2-4] and identifying the right eye as site of magnetoreception [5]. The initial step of the processes leading to magnetoreception, the absorption of a photon by a suitable photopigment, makes the detection of magnetic directions light-dependent and raises the question of how magnetoreception is affected by different light regimes.

The light-dependency of magnetoreception was analyzed in a series of experiments using migratory orientation of passerines as a criterion whether or not birds could derive directional information from the magnetic field in a given situation. The test lights were monochromatic lights produced by LEDs (light-emitting diodes) with a bandwidth in the range of 30 to 50 nm at half the maximum intensity. Tests with Australian silvereyes, *Zosterops l. lateralis*, [6,7], European robins, *Erithacus rubecula *[8-10] and European garden warblers, *Sylvia borin *[11] revealed that magnetoreception requires light from the blue-to-green part of the visual spectrum: the birds were well oriented in their seasonally appropriate migratory direction under wavelengths up to 565 nm green light, whereas they were disoriented under 590 nm yellow and beyond. In addition to these passerines, a similar wavelength-dependency is indicated for homing pigeons [12], so that it can be assumed to be rather widespread among birds.

The initial tests with migrants were performed under the rather low light intensity of 6 to 9·10^15 ^quanta s^-1^m^-2^, a light level found in nature more than half an hour before sunrise or after sunset. When the light intensity was increased sevenfold, which still corresponds to light levels before sunrise or after sunset, a surprising phenomenon became evident: while the birds continued to be disoriented under yellow and red light [10,13], they ceased to prefer their migratory direction as they had done before under the blue-to-green range of the spectrum. Instead, they showed axial headings along the east-west axis or occasionally unimodal tendencies in 'fixed' directions that did not change between spring and autumn [4,7,10,14]. These were unexpected findings. During migration season, birds are highly motivated to head into their migratory direction; hence their altered behavior implies that the magnetic compass system was disrupted and could no longer provide the directional information required to locate the migratory direction – brighter monochromatic lights seem to interfere with magnetoreception.

The nature of the observed responses is unclear and raises the question about the factors controlling this behavior. Exposing European robins to a broad-band oscillating magnetic field (0.1 to 10 MHz) indicated that their orientation under high intensity turquoise light was no longer based on the radical pair mechanism underlying the normal magnetic compass [4]. A case that might involve a similar phenomenon has been described in amphibians: after pre-treatment with certain light regimes, salamanders showed an axial preference that in some animals was associated with the orientation of magnetite crystals in their heads [15]. So it seemed possible that the behavior of the robins under monochromatic light of higher intensities was no longer controlled by light, but instead by magnetite or magnetite-based receptors.

As a first step to test this hypothesis, we performed a systematic study on the orientation of European robins under monochromatic light of different intensities under blue, turquoise and green light, with additional tests under UV. If the birds' responses were still controlled by a light-dependent mechanism, one would predict that a further increase in light intensity should continue to affect their behavior. – Our findings show that this is indeed the case.

## Results

The robins were tested in spring under 424 nm Blue, 502 nm Turquoise and 565 nm Green at four different intensities. The tests under a quantal flux of 8·10^15 ^quanta s^-1 ^m^-2^, a light level where birds had always shown good orientation in migratory direction, served as controls. The other test intensities were 36, 54 and 72·10^15 ^quanta s^-1 ^m^-2^. Under these higher light levels, we observed a phenomenon that rarely occurred under dim light: the behavior became axially bimodal, with the birds preferring a direction and it's opposite. This was true for the distribution of activity of individual recordings as well as for the headings of individual birds. To take this axiality into account, we used in case of axiality the preferred end of the axes for further analysis (for details, see method section).

**Figure 1:**
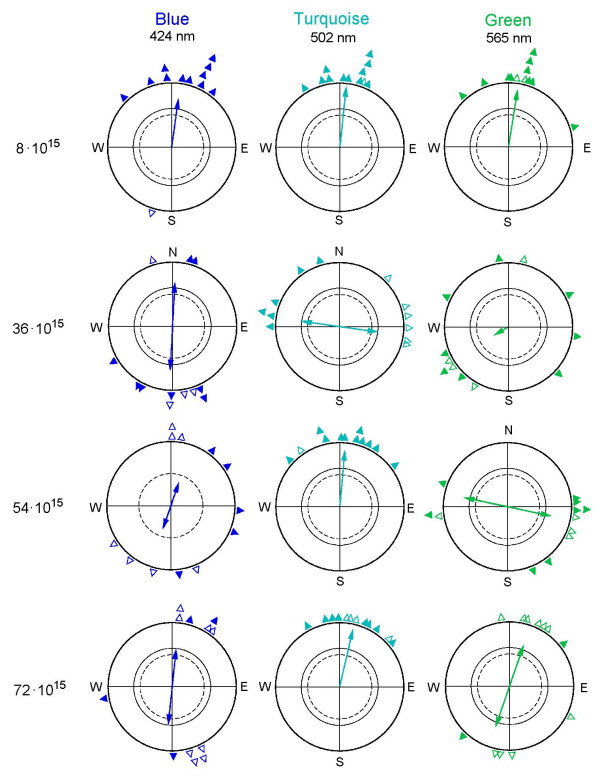
**Orientation of European robins under 424 nm Blue, 502 nm Turquoise and 565 nm Green light of different quantal flux **(given on the left side in quanta s^-1^m^-2^). The triangles at the periphery of the circles mark the mean headings of individual birds, with solid symbols indicating unimodal means and open symbols indicating the preferred end of mean axes (see text). The arrows and double arrows represent the grand mean vectors and grand mean axes, respectively, with the two inner circles marking the 5% (dotted) and 1% significance border of the Rayleigh test [47].

Fig. 1 gives the individual birds' mean headings and the grand mean vectors or axes. The latter are given numerically in Table 1, together with the median of the individual birds' vector lengths. The mean vectors or mean axes, respectively, of the individual birds are given in Table 2.

**Table 1:** Orientation data of three groups of 12 birds, recorded under various intensities of blue, turquoise or green light

	424 nm Blue					502 nm Turquoise					565 nm Green				
			
Intensity	axr	axb	med r_b_	α_N_	r_N_	axr	axb	med r_b_	α_N_	r_N_	axr	axb	med r_b_	α_N_	r_N_
8·10^15 ^qu/s m^2^	11	17	0.92	8°	0.76***	6	8	0.94	5°	0.96***	8	17	0.89	9°	0.91***
36·10^15 ^qu/s m^2^	17	33	0.61	3°–183°	0.69**	50	50	0.96	98°–278°	0.61**	19	33	0.72	(55°–235°)	0.27^n.s.^
54·10^15 ^qu/s m^2^	31	58	0.73	(19°–199°)	0.39^ns^	8	8	0.92	5°	0.89***	28	33	0.70	102°–282°	0.70**
72·10^15 ^qu/s m^2^	25	67	0.77	6°–186°	0.60**	6	33	0.85	13°	0.93***	19	83	0.88	19°–199°	0.68**

axr, axb, percentage of axial recordings, and axial mean vectors of birds, respectively (both as defined in text); med r_b_, median vector length of the individual birds, representing the intra-individual variance; α_N_, r_N_, grand mean vector or axis (non-significant axes in parentheses), with asterisks at r_N _indicating significance by the Rayleigh test: n.s., not significant; * *P *< 0.05; ** *P *< 0.01 and *** *P *< 0.001.

**Table 2:** Vectors of individual birds based on 3 recordings each

	8·10^15 ^qu s^-1^m^-2^		36·10^15 ^qu s^-1^m^-2^		54·10^15 ^qu s^-1^m^-2^		72·10^15 ^qu s^-1^m^-2^	
				
Bird	α_b_	r_b_	α_b_	r_b_	α_b_	r_b_	α_b_	r_b_
*424 nm*	*Blue*							
03-1	25°	0.98	343°	0.63^A^	9°	0.84^A^	33°	0.98
03-2	342°	0.85	182°	0.74^A^	158°	0.83^A^	154°	0.78^A^
03-3	37°	0.97	156°	0.66	195°	0.94^A^	179°	0.59
03-4	356°	0.92	207°	0.43	173°	1.00	165°	0.65^A^
03-5	9°	0.94	181°	0.81	37°	0.64	6°	0.98^A^
03-6	197°	0.62^A^	210°	0.38	94°	0.70	15°	0.99
03-7	24°	0.91	162°	0.95^A^	1°	0.75^A^	36°	0.35^A^
03-8	26°	0.96	15°	0.29	113°	0.62	6°	0.86^A^
03-9	316°	0.80	170°	0.48^A^	236°	0.66^A^	163°	0.75^A^
03-10	355°	0.92	239°	0.47	1°	0.68^A^	260°	0.36
03-11	25°	0.70^A^	156°	0.74	217°	0.53^A^	32°	0.86^A^
03-12	15°	0.91	19°	0.58	55°	0.89	156°	0.68^A^
*502 nm*	*Turquoise*							
02-1	345°	0.91	279°	0.97	53°	0.71	6°	0.80^A^
02-2	17°	0.59	81°	0.98^A^	325°	0.35^A^	33°	0.82
02-3	7°	0.95	270°	0.99	1°	0.99	348°	0.75
02-4	18°	0.96	105°	0.98^A^	15°	0.92	46°	0.66^A^
02-5	20°	0.76^A^	45°	0.51^A^	32°	0.98	14°	0.87^A^
02-6	329°	1.00	91°	0.98^A^	25°	0.91	50°	0.90
02-7	350°	0.86	291°	0.93	345°	0.99	9°	0.83^A^
02-8	19°	0.97	281°	0.99	19°	0.96	354°	0.80
02-9	17°	0.67	343°	0.71	4°	0.94	22°	0.96
02-10	24°	0.95	102°	0.67^A^	14°	0.90	25°	0.98
02-11	3°	0.93	73°	0.94^A^	348°	0.81	332°	0.90
02-12	351°	0.96	325°	0.29	313°	0.61	359°	0.94
*565 nm*	Green							
04-1	339°	0.81	234°	0.78^A^	99°	0.20	223°	0.54
04-2	2°	0.92	240°	0.57^A^	290°	0.71	12°	0.96^A^
04-3	14°	0.82^A^	13°	0.34^A^	83°	0.99	353°	0.98^A^
04-4	7°	0.82^A^	247°	0.68	116°	0.68^A^	178°	0.92^A^
04-5	20°	1.00	62°	0.64	143°	0.67	30°	0.94^A^
04-6	14°	0.86	223°	0.80	93°	0.99^A^	15°	1.00^A^
04-7	73°	0.58	210°	0.69^A^	86°	0.95	188°	0.81^A^
04-8	15°	0.98	98°	0.93	111°	0.97^A^	25°	0.84^A^
04-9	360°	0.97	296°	0.66	264°	0.65	52°	0.66
04-10	17°	0.94	235°	0.74	158°	0.52	117°	0.37^A^
04-11	318°	0.56	352°	0.86	262°	0.38^A^	34°	0.84^A^
04-12	14°	0.99	133°	0.77	92°	0.83	192°	0.95^A^

^A ^indicates axial vectors, with the preferred end of the axis indicated under α_b_

Under the low Blue, Turquoise and Green light of 8·10^15 ^quanta s^-1 ^m^-2^, the birds headed in their seasonally appropriate spring migratory direction slightly east of North. These three distributions are not different from each other (*P *> 0.05), with long vectors, indicating excellent agreement among the 12 birds tested.

Under brighter light, the behavior depended on the wavelength as well as on the intensity of light:

(1) Under 424 nm Blue, the birds preferred the north-south axis in all three remaining intensities. The axial preferences are significant for 36 and 72·10^15 ^quanta s^-1 ^m^-2^; the axis observed under 54·10^15 ^quanta s^-1 ^m^-2^, although not significant, has a considerable length and suggests a similar tendency. The three distributions are not significantly different from each other (*P *> 0.05). – In another series performed in autumn under 424 nm Blue at 30·10^15 ^quanta s^-1 ^m^-2^, slightly lower than the 36·10^15 ^quanta s^-1 ^m^-2 ^used in spring, robins showed a significant preference for the east-west axis (n = 16, 98°–278°, r_N _= 0.82, *P *< 0.001)

(2) Under 502 nm Turquoise, the birds preferred the east-west axis under 36·10^15 ^quanta s^-1 ^m^-2 ^and showed unimodal northerly tendencies at the two higher intensities. The axial preference is significantly different (at least *P *< 0.01) from the unimodal headings which do not differ from each other (*P *> 0.05).

(3) Under 565 nm Green, the most diverse behavior was observed, with all three samples significantly differing from each other (at least *P *< 0.05): disorientation under 36·10^15 ^quanta s^-1 ^m^-2^, a preference for the east-west axis under 54·10^15 ^quanta s^-1 ^m^-2 ^and a preference for the north-south axis under 72·10^15 ^quanta s^-1 ^m^-2^.

Table 1 also includes the percentage of axial recordings, which was low under the low intensity of the three colors and also under Turquoise where the birds showed unimodal headings, but markedly higher in the other conditions. A similar relationship is found with the percentage of axial vectors of individual birds, which reached more than 50% in some cases when the birds showed axial preferences. The median vector lengths of the individuals, reflecting the intra-individual variance, were fairly high in all conditions.

**Figure 2:**
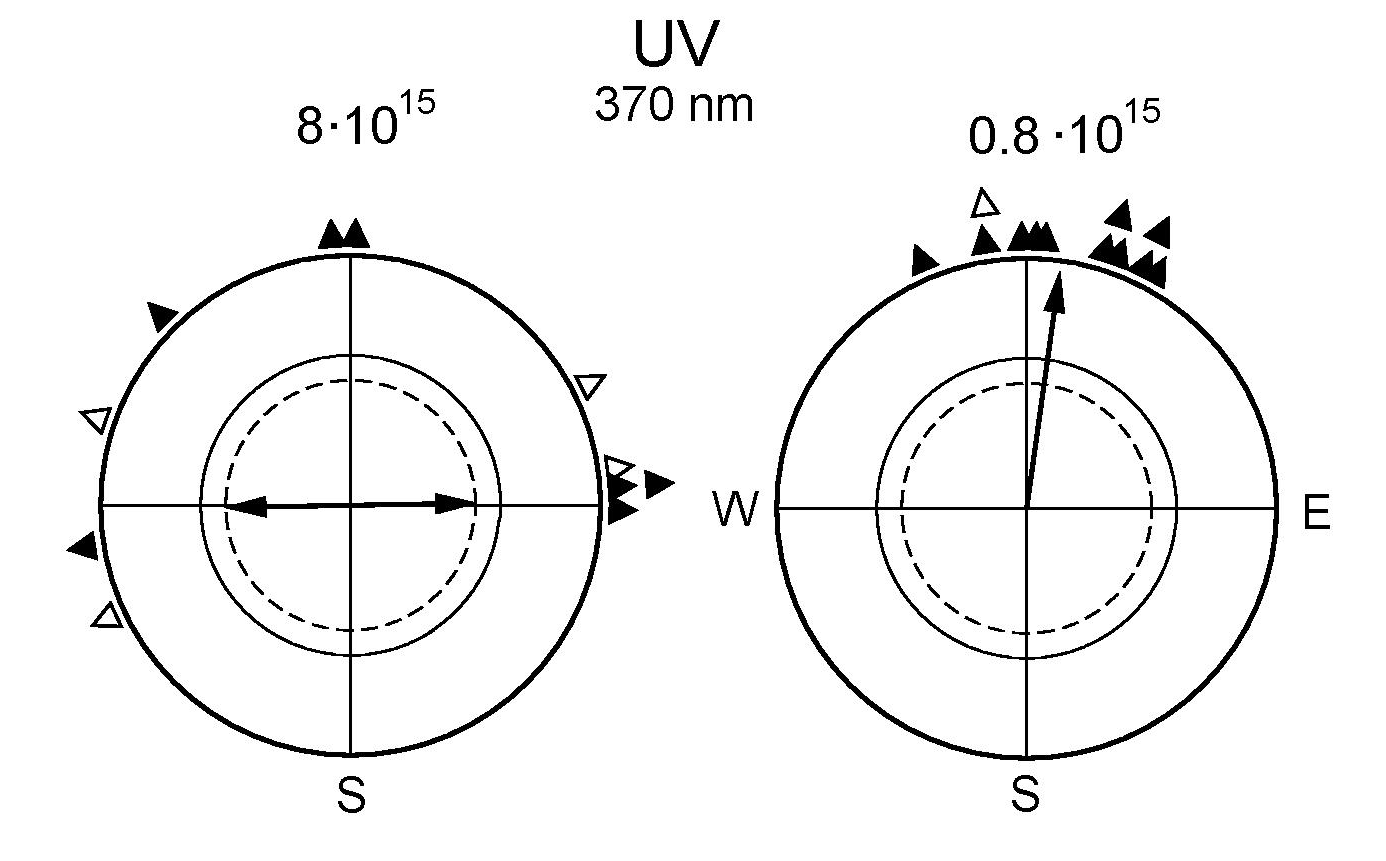
**Orientation of European robins under 373 nm UV light of different quantal flux **(in quanta s^-1^m^-2 ^above the circle). Symbols as in figure 1.

In autumn, we also performed tests under 373 nm UV at 8·10^15 ^quanta s^-1 ^m^-2^, which is the light level that represented the lowest intensity in the above-mentioned experiments. We observed an axial preference for the east-west axis (n = 16; 74°–254°, r_N _= 0.58, *P *< 0.01). The results of the following spring experiments under this light regime again produced an axial preference for the east-west axis under 8·10^15 ^quanta s^-1 ^m^-2 ^(Fig. 2, left, and Table 3), which looked similar to the respective distributions under Blue, Turquoise and Green at higher intensities. However, in tests under 373 nm UV at 0.8·10^15 ^quanta s^-1 ^m^-2^, i.e. at only one tenth of the quantal flux, the robins preferred their seasonally appropriate northerly migratory direction (Fig. 2, right). The directional choices of the individual birds are given in Table 4.

**Table 3:** Orientation of 12 birds recorded under UV light of two intensities

	373 nm UV				
	
Intensity	axr	axb	med r_b_	α_N_	r_N_
8·10^15 ^qu/s m^2^	47	50	0.76	89°–269°	0.50*
0.8·10^15 ^qu/s m^2^	11	8	0.95	8°	0.96***

axr, axb, percentage of axial recordings and axial mean vectors of birds, respectively (both as defined in text); med r_b_, median vector length of the individual birds, representing the intraindividual variance; α_N_, r_N_, grand mean vector or axis (non-significant data in parentheses), with asterisks at r_N _indicating significance by the Rayleigh test, see Table 1

**Table 4:** Vectors of individual birds under 373 nm UV light based on three recordings each

	8·10^15 ^qu s^-1^m^-2^		0.8·10^15 ^qu s^-1^m^-2^	
		
Bird	α_b_	r_b_	α_b_	r_b_
01-1	86°	0.90	26°	0.75
01-2	261°	0.97	29°	0.99
01-3	86°	0.78^A^	26°	1.00
01-4	356°	0.71	18°	0.91
01-5	289°	0.29^A^	359°	0.95
01-6	315°	0.90	20°	0.95
01-7	63°	0.57^A^	17°	0.93
01-8	91°	1.00^A^	351°	0.66
01-9	86°	0.97	337°	0.59
01-10	246°	0.58^A^	4°	0.98
01-11	82°	0.55^A^	2°	0.98
01-12	1°	0.72	352°	0.98^A^

^A ^indicates axial vectors, with the preferred end of the axis indicated under α_b_

## Discussion

The orientation responses observed under UV, Blue, Turquoise and Green light at the various intensities clearly fall into two distinct categories: under the lowest light level, which was 0.8·10^15 ^quanta s^-1 ^m^-2 ^for UV and 8·10^15 ^quanta s^-1 ^m^-2 ^for Blue, Turquoise and Green, the robins showed a strong preference for their migratory direction. Under 8·10^15 ^quanta s^-1 ^m^-2 ^UV light and under increased light intensities of the other colors, a variety of responses, mostly axial preferences, was observed. The northerly headings observed under bright Turquoise, although superficially similar to the migratory direction, also represent responses of a different nature [4] (see below). Together, the data clearly show that even when the birds were no longer heading in their migratory direction, their behavior continued to change as the light intensity increased. This implies that it is still controlled by light-dependent processes.

The following considerations will focus on (i) the intensity-dependent pattern of responses indicated, (ii) possible reasons for the change in the nature of the responses, (iii) the possible involvement of the color cones and (iv) the origin of directional information for the axial and 'fixed direction' responses.

### Compass orientation and other responses

The behavior under the low light intensity Blue, Turquoise and Green represent true migratory orientation: under this and similar light levels, birds prefer their migratory direction in spring as well as in autumn. They showed the expected seasonal reversal, and the compass mechanism involved is the normal avian inclination compass [4,7,16]. Tests with oscillating magnetic fields indicate that magnetoreception under Green and Turquoise light is based on radical pair processes [2-4] as proposed by the model of Ritz and colleagues [1]. The same can be assumed for dim Blue light, where the orientation corresponds to that under Green and Turquoise in all other aspects [17], and presumably also for UV light of very low intensity. That is, under low monochromatic lights from 373 nm UV to 565 nm Green, magnetoreception works in the normal way as under 'white' light, providing birds with directional information from the magnetic field that they can use to locate their migratory direction and probably also any other direction they may wish to pursue.

The behavior under monochromatic light of higher intensity is different. Increased monochromatic lights do not simply cause a switch from migratory orientation to another specific response; instead, they elicit a variety of different responses. This is most conspicuous under green light: Here, the birds show disorientation at 36·10^15 ^quanta s^-1 ^m^-2^, then, as intensity increases, a preference for the east-west axis and finally a preference for the north-south axis. Muheim and colleagues [18], also testing robins under green light, observed axial behavior in the migratory direction and in the opposite direction under intensities of 14 and 29·10^15 ^quanta s^-1 ^m^-2^, which may be a first step away from normal migratory orientation.

Together, the responses observed under 8·10^15 ^quanta s^-1 ^m^-2 ^UV and at higher intensities of Blue, Turquoise and Green suggest that the pattern observed under Green might be a general one. Under all wavelengths, we found a preference of the east-west axis that, under increased intensity, was followed by a preference of the north-south axis, with the modification that under Turquoise, a unimodal 'fixed' northerly direction [4] replaces the axial north-south tendency. However, where normal migratory orientation ends and random and axial behavior begins appear to depend on the wavelength as well as on the intensity of light.

The different types of responses at brighter light imply a disrupted function of the magnetoreception system under monochromatic light of higher intensity. It raises a number of questions: What causes the magnetoreception system to cease functioning in the normal way? Is there a functional significance of the axial and 'fixed' direction responses? And: what is the nature of the directional information for the 'fixed' directions and axial responses?

### What causes the change in magnetoreception?

An effect of the brighter monochromatic lights on circadian patterns and motivation (e.g. [19,20]) is rather unlikely in view of the fact that even the brightest lights used in the present study were of intensities found well after sunset (see Method section), i.e., at a time of day when nocturnal migration is in progress. Also, the disruption of the normal magnetic perception process cannot be attributed to the higher intensity of light itself or to saturation of the crucial receptors. The avian magnetic compass works under bright sun light (see e.g. [21-24] for homing pigeons and day migrants), and cage tests showed that also nocturnal migrants use their magnetic compass for migratory orientation under natural day light when migratory behavior was induced by food deprivation [25]. Caged robins, too, were well oriented under 'white' test lights of higher intensity [13]. However, the 'white' test lights, like day light, were composed of wavelengths from all parts of the spectrum. Therefore, it seems to be the narrow bandwidth of the monochromatic test lights used rather than their brightness that gives rise to the observed effects.

The same wavelengths of light allow very good orientation at low intensities, but disrupt the magnetic compass orientation as the intensity of the monochromatic light increases. Our experiments were performed under light levels that, in humans, are mesopic conditions, i.e., where both the rod and the cone system is active. In humans, this transition zone covers at least 3 log units [26]; its extension in birds is unknown. Note that the light levels where we observed a change from compass orientation to an axial response along the east-west axis increased with increasing wavelengths, from UV over Blue and Turquoise to Green, suggesting a similar relationship for the end of normal perception of magnetic directions. This is a striking parallel to the sensitivity of the color cones, which decreases with increasing wavelength, with the UV-sensitive-cone type being more sensitive than the short-wavelength-sensitive cone, this type being more sensitive than the medial-wavelength-sensitive cone and the long-wavelength-sensitive cone type being least sensitive [see e.g. [27]]. This implies an involvement of the color cones under the higher light intensities, suggesting that the perception of magnetic directions works properly under monochromatic light as long as the test lights do not activate the cones above a certain level.

### A possible role of the color cones?

The radical pair model [1] proposes that photon absorption causes a photopigment to form radical pairs and generate the signals that mediate magnetic compass information. However, at present, neither the nature of the relevant photopigment nor the type of cells where the reception processes take place are precisely known. A role of the rods and color cones in avian magnetoreception is usually not considered, because rhodopsin and the other opsins do not form the required radical pairs. Ritz et al. [1] therefore proposed that these could be formed by cryptochrome, a novel photopigment first known from plants (see [28]), but also found in the retina of chickens [29] and of passerine birds [30,31]. Cryptochromes have recently been shown to mediate magnetic effects in plants [32]. This implies the possibility of specialized photoreceptors for magnetoreception. The disruptive effect of intense monochromatic light, on the other hand, suggests that these receptors may interact with the normal visual perception system, in some complex way.

One possibility is that cryptochrome does not directly absorb the light, but receives the energy from a light-harvesting system of other pigments, as it is proposed, e.g., for photosynthesis [33]. Yet under this assumption, it is hard to explain why higher light intensities should lead to a change in the nature of response away from normal compass orientation to axial responses and 'fixed direction'.

However, the behavior suggesting an involvement of the cones is only observed under higher intensity monochromatic light. This has to be included in the considerations on the role of cones in magnetic perception. The answer may lie in the fact that the output of a given cone is affected by both, the wavelength of the incident light and its intensity. Both parameters together give only one output value. Color perception is then based on the balance of the outputs of the three (mammals) or four (birds) cone types, as it is measured, for example, by the retinal ganglion cells where the input from the photoreceptors converges. Natural light will always excite several types of cones, since even objects that appear unicolored to us usually reflect a multitude of different wavelengths. Hence all cone types normally receive at least a certain amount of excitation by photons. In view of this, it is quite conceivable that the color system is tuned to perceive a mixture of almost all visual wavelengths under normal conditions.

Monochromatic light would cause an imbalance between the different receptors, and there is a lot of evidence that a strong imbalance in the color of the visual scene lead to strong habituation of selected cones, which in turn causes the appearance of aftereffects like the sensation of the countercolor when the imbalance is eliminated [34]. By using monochromatic light with only a narrow spectral band, but of a relatively high intensity, the difference between the excitation of the cones projecting to one opponent color ganglion cell might become too large to be accepted by the system as normal, and the ganglion cell will no longer produce the appropriate activity. This may cause the visual system to also reject the magnetic information because it could be erroneous. In other words, the visual system may be able to gate, i.e. control the transfer, of the magnetic input somewhere on its way to the brain area where it is processed. Although there is ample evidence for the existence of such gating systems – almost all sensory information, for example, is thought to be gated in the thalamic nuclei on its way to the forebrain [35] – this is a mere assumption in the case of magnetic information. The activation of a visual brain area only at night recently described [36] might be an example of a gating process that allows the transfer of information towards this area only under certain conditions.

Whether an imbalance between the different color receptors is the correct explanation for the responses observed under high intensity monochromatic light must remain open at present. It means, however, that these responses need not necessarily be of functional significance. Instead, they might be by-products of a perception system driven beyond its functional limits, reflecting a complex relationship between various receptors and units that awaits further analysis.

### Where does the polar magnetic information originate?

The unimodal response at 54·10^15 ^quanta s^-1 ^m^-2 ^Turquoise was found to be polar, not involving the normal avian inclination compass, and tests applying high frequency fields showed that it is not based on radical pair processes [4]. It seems likely that the axial responses under intense monochromatic light share these characteristics. This raises the question where this type of directional information comes from, if it does not originate in radical pair processes.

A magnetite-based receptor seems to be a logical assumption, as magnetite-based receptors could convey polar directions (see e.g. [37]). Magnetite has been found in the ethmoid region and in the upper beak of birds [38,39], but electrophysiological recordings from the corresponding branch of the trigeminal nerve [40] as well as behavioral studies [41-43] seemed to suggest that magnetite-based receptors in birds provide information on magnetic intensity rather than directional information. However, it cannot be excluded that they additionally mediate directional information. The relationship between the axial preference and the orientation of magnetite particles described in salamanders [15] appears to suggest a role of magnetite in these responses, and a recent study [44] indicates that another 'fixed' direction response in birds was indeed mediated by the iron-based receptors in the upper beak [39].

Although a magnetite-based mechanism is certainly an option, it must be considered that the specific manifestation of the behavior observed under monochromatic light of higher intensity clearly depends on the intensity and wavelength of light. This is not only true for the present study, but also for various 'fixed' directions observed in other studies [7,14,17,45]. The control of the axial and 'fixed' direction responses by the ambient light regime is difficult to explain by attributing it to a magnetite-based mechanism without auxiliary assumptions, like e.g. interaction between the magnetite-based and light-dependent mechanisms.

## Conclusion

Our study with monochromatic Blue, Turquoise, Green and UV light revealed two distinct types of orientation behavior depending on the intensity of light: normal compass orientation under very low light, and a variety of different responses under light of higher intensity, probably changing according to a specific pattern as intensity increases. The occurrence of these responses indicates a complex interaction of various receptors and for the first time suggests an involvement of the color cone system in magnetoreception.

The interpretation of our findings suffers greatly from the fact that the cells representing the magnetoreceptors have not yet been reliably identified (but see [1,31]) and that, as a consequence, we do not know how they are interconnected with other units. In general, we know too little about the wiring of the avian retina, especially the color processing system, to come up with an explanation that is not entirely speculative. As to a possible involvement of magnetite-based receptors in the axial and 'fixed' direction responses, future tests will have to clarify their role.

## Materials and methods

The experiments reported here were performed in the garden of the Zoological Institute in Frankfurt a.M., Germany (50°08'N 8°40'E) in pre-spring during a six week period each from the first week of January to about mid-February.

### Test birds

The test birds were European robins, a night-migrating passerine species. Groups of 12 robins each were mistnetted as transmigrants in the Botanical garden in Frankfurt during the first two weeks of September of the year before the tests. The birds were juveniles identified as being of Scandinavian origin by their relatively long wings. They were kept indoors in individual cages under white light from a fluorescent lamp in a photoperiod that simulated the natural one outside until the beginning of December, when their photoperiod was decreased to L:D 8:16. Around New Year, it was changed in two steps to L:D 13:11. This induced premature spring migratory restlessness so that the tests could begin in the first week of January. During testing and thereafter, the photoperiod was maintained at L:D 13:11, and in the last week of March, when this photoperiod was reached outside, the birds were released at the site of capture.

### Test lights

**Figure 3:**
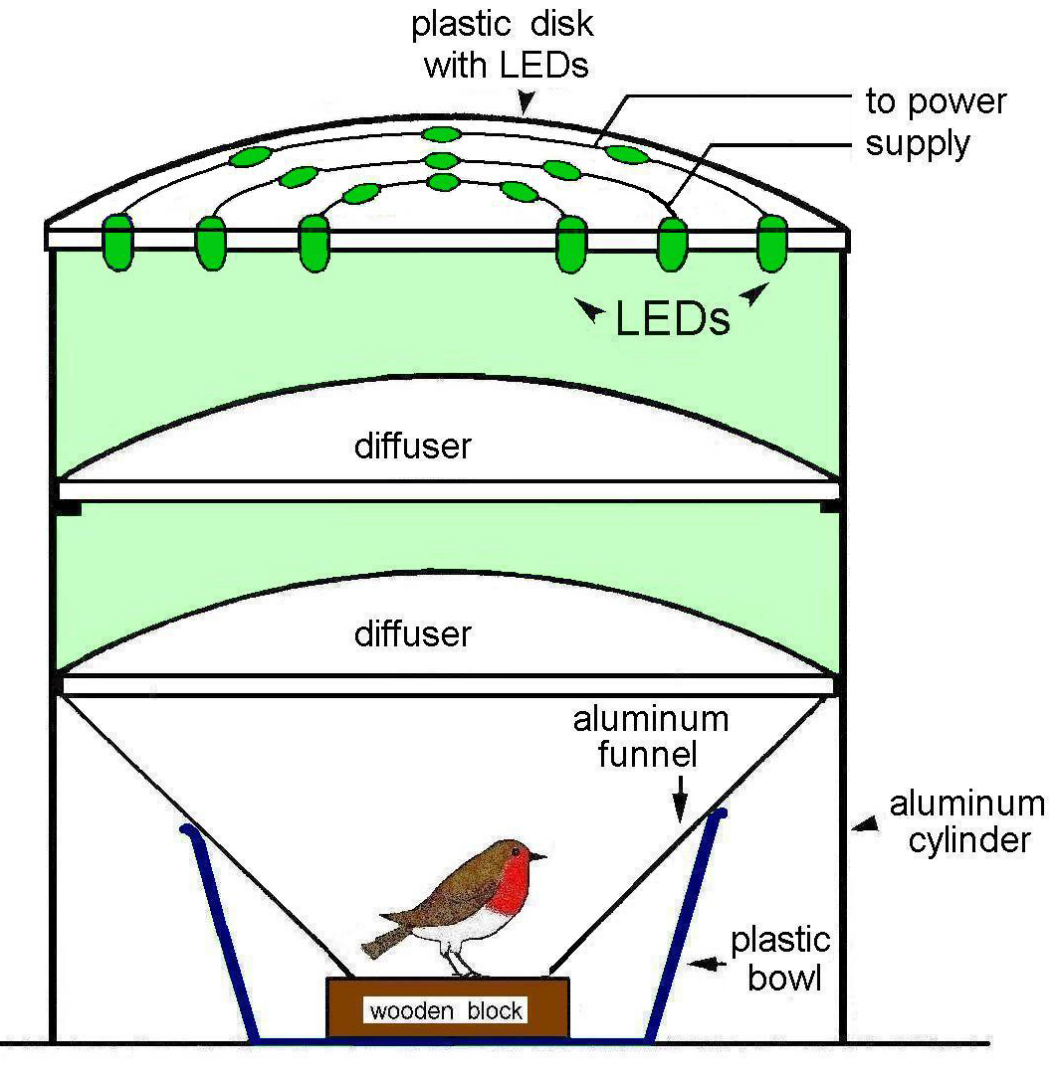
Test apparatus for testing individual birds under monochromatic lights.

The test lights were produced using the same (Blue, Green) or similar (Turquoise) LEDs (light-emitting diodes) as in earlier studies (e.g. [7,10]). Their spectra and that for UV used here for the first time are given in Table 5. Sets of 24 or 48 LEDs, or, for UV, of 3 LEDs, mounted on a plastic disc were suspended above the test cages (see Fig. 3), with the light intensities controlled by adjusting current and the numbers of LEDs activated to produce test lights of equal quantal flux. The four light levels were about 8, 36, 54 and 72·10^15 ^quanta s^-1 ^m^-2^, for Blue, Turquoise and Green and 8 and 0.8·10^15 ^quanta s^-1 ^m^-2 ^for UV. The blue to green lights were measured in the test cages as irradiance using Optometer P9710-1 (Gigahertz-Optik, Puchheim, Germany) with the radiometric probe "Visible" RW-3703-2, a silicon photoelement for the wavelength range 400 – 800 nm, and the UV light with the corresponding UV probe. The light levels used here are generally slightly higher than the respective ones used in the previous studies (e.g. [7,10]).

**Table 5:** Emission spectra of the LEDs used in the present paper

Color	Peak wavelength	50% intensity (low, high)
UV	373 nm	(368 nm, 381 nm)
Blue	424 nm	(403 nm, 459 nm)
Turquoise	502 nm	(486 nm, 518 nm)
Green	565 nm	(553 nm, 583 nm)

For an idea what the four light levels mean compared with the natural light outside, let us state that e.g. the green light corresponded to light found outside under largely clear sky more than 45 min, about 38 min, 34 min and 32 min before sunrise and after sunset, or, if only the green part of the spectrum between 553 and 583 nm is considered, to light about 28 min, 20 min, 17 min and 15 min before sunrise and after sunset (estimates based on our own measurements).

### Test apparatus and performance

The tests were performed in wooden huts in the garden of the Zoological Institute, where the local geomagnetic field was undisturbed with an intensity of 46 000 nT, and + 66° inclination. The directional tendencies of the birds were recorded in funnel cages [46] lined with coated paper (typewriter correction paper BIC, Germany; formerly Tipp-Ex), where the birds were tested one at a time (see [6]). Each funnel cage was placed inside an aluminum cylinder, which isolated the cages against each other, with the top of the cylinder consisting of the plastic disk carrying the LEDs (Fig. 3). The light passed through two sets of diffusers before it reached the test bird.

Recording the robins' orientation began in the evening at about the time when the light went off in the housing cages and lasted for 75 min. When active, the birds left scratch marks on the coating of the inclined walls of the cages, which documented the distribution of their activity. Each bird was tested under the various test conditions until they had produced three recordings with sufficient activity (= 35 scratches) in each.

### Data analysis

After removal from the cage, the coated paper was divided into 24 sectors, and the scratch marks in each sector were counted. From the distribution of the scratches, the bird's heading and the concentration of activity (expressed by the vector length) of the respective test were calculated. From the three headings of each bird, we calculated the mean vector of this bird under each condition, with direction α_b _and length r_b_. Based on the mean headings α_b _of the 12 test birds, the grand mean vector for each condition, with the direction α_N _and the length r_N_, was calculated.

Under higher light levels, the birds often showed axial behavior, with the scratches within the cage bimodally distributed along an axis, as indicated by a higher concentration (longer vector) obtained when the angles were doubled (modulo 360°) so that opposite sectors fall together [47]. At the same time, many birds' vectors r_b _increased considerably when they were calculated by doubling the angles (modulo 360°), indicating axial choices with two of the three headings on one side and one at the other. To take this axiality adequately into account, we followed the procedure used in [[48], [49]]: all recordings with the axial vector at least 0.03 longer than the respective unimodal vector were treated as axial. Likewise, when the three recordings of each bird were comprised, the mean vector as well as the mean axis was calculated, and the bird's behavior was considered axial when the axial vector length r_b _was at least 0.10 longer than the unimodal vector length r_b_. In these cases, we used the preferred end of the axis (i.e. in case of the single recordings, the end with more activity and, in case of a bird's axial vector, the end with more headings, expressed by its being closer to the direction of the unimodal vector) for further calculations. The data from all test conditions are treated this way, and the percentage of axial recordings as well as the percentage of axial bird vectors r_b _is included in Table 1. When calculating the grand mean vectors from the mean headings of the individual birds, α_b_, we also calculated the grand axis by doubling the angles to test for an axial distribution of means, and used the longer vector for deciding between unimodal and axial preferences.

### Statistical analysis

The grand mean vectors or grand axes were tested by the Rayleigh test for directional preferences [47]. The birds' mean headings under the low light levels were compared with the parametric Watson Williams test for differences in direction. Within each color, the distribution of the mean headings was compared with the non-parametric Mardia Watson Wheeler test [47]. Since here most vectors were axial, we applied this test to the transformed distributions resulting from doubling the angles (modulo 360°). From the vector lengths r_b _per bird (considering the axial vector length when the vectors was defined as axial), we calculated the grand medians.

## Competing interests

The author(s) declare that they have no competing interests.

## Authors' contributions

RW designed the study, did the statistical analysis and wrote the manuscript. KS took a large part in providing the test birds and performing the experiments, in particular in controlling the various light regimes. HJB provided expertise on the avian visual system and wrote part of the discussion. WW contributed significantly to designing the study, had designed test apparatus and testing procedure, had a major part in the data analysis and modified the manuscript.
